# A prognostic tool to identify adolescents at high risk of becoming daily smokers

**DOI:** 10.1186/1471-2431-11-70

**Published:** 2011-08-11

**Authors:** Igor Karp, Gilles Paradis, Marie Lambert, Erika Dugas, Jennifer O'Loughlin

**Affiliations:** 1 University of Montréal Hospital Research Centre (CRCHUM), Montreal, Quebec, Canada; 2 Department of Social and Preventive Medicine, University of Montréal, Montreal, Quebec, Canada; 3 Department of Epidemiology, Biostatistics and Occupational Health, McGill University, Montreal, Quebec, Canada; 4 McGill University Health Center Research Institute, Montreal, Quebec, Canada; 5 Institut national de santé publique du Québec, Montreal, Quebec, Canada; 6 Centre de recherche de CHU Ste-Justine, Montreal, Quebec, Canada; 7 Department of Pediatrics, University of Montreal, Montreal, Quebec, Canada

## Abstract

**Background:**

The American Academy of Pediatrics advocates that pediatricians should be involved in tobacco counseling and has developed guidelines for counseling. We present a prognostic tool for use by health care practitioners in both clinical and non-clinical settings, to identify adolescents at risk of becoming daily smokers.

**Methods:**

Data were drawn from the Nicotine Dependence in Teens (NDIT) Study, a prospective investigation of 1293 adolescents, initially aged 12-13 years, recruited in 10 secondary schools in Montreal, Canada in 1999. Questionnaires were administered every three months for five years. The prognostic tool was developed using estimated coefficients from multivariable logistic models. Model overfitting was corrected using bootstrap cross-validation. Goodness-of-fit and predictive ability of the models were assessed by R^2^, the c-statistic, and the Hosmer-Lemeshow test.

**Results:**

The 1-year and 2-year probability of initiating daily smoking was a joint function of seven individual characteristics: age; ever smoked; ever felt like you needed a cigarette; parent(s) smoke; sibling(s) smoke; friend(s) smoke; and ever drank alcohol. The models were characterized by reasonably good fit and predictive ability. They were transformed into user-friendly tables such that the risk of daily smoking can be easily computed by summing points for responses to each item. The prognostic tool is also available on-line at http://episerve.chumontreal.qc.ca/calculation_risk/daily-risk/daily_smokingadd.php.

**Conclusions:**

The prognostic tool to identify youth at high risk of daily smoking may eventually be an important component of a comprehensive tobacco control system.

## Background

Despite considerable declines in prevalence, cigarette smoking remains the leading avoidable threat to the health of children and adolescents. In 2006-7, nearly 50,000 Canadian youth in grades 5-9 were current smokers[1]. Further, the steady decline in the prevalence of youth smoking over the past decade has now leveled off,[2] suggesting that continued concerted effort to control cigarette smoking is needed.

Cigarette smoking usually begins during early adolescence and it is now known that nicotine dependence (ND) symptoms can develop soon after first puff[3]. Withdrawal symptoms in particular present a serious obstacle to quitting and although the desire to quit can begin soon after smoking onset,[3] the majority of youth fail in their quit attempts[4]. Daily smoking is a particularly strong risk factor for the development of cravings, withdrawal symptoms and tolerance in adolescents, to the extent that prevention of daily smoking may represent a pivotal disease prevention strategy[5].

The American Academy of Pediatrics advocates that pediatricians should be involved in tobacco counseling and has developed guidelines for counseling by pediatricians[6]. However, according to a recent survey, less than half of general practitioners in Montreal advised their young patients not to smoke, and only one-third felt that they had the skills to prevent their young patients from starting to smoke[7]. Youth smoking interventions in clinical settings are therefore paradoxically both acknowledged as important and currently not widely implemented.

Limited time is likely an important perceived barrier to tobacco counselling among busy clinicians. Similar to prevention counselling for cardiovascular diseases in adults based on the Framingham equations,[8,9] youth smoking counselling may be facilitated if it were possible to accurately assess the risk of becoming a sustained smoker. Youth whose risk is high could then be selectively targeted for intensive intervention. We present the development of a prognostic tool for use by health care practitioners, to identify adolescents at risk of becoming daily smokers.

## Methods

Data for this analysis were drawn from the Nicotine Dependence in Teens (NDIT) Study,[10] an ongoing prospective cohort investigation of 1,293 students initially aged 12-13 years recruited from grade 7 classes in a convenience sample of 10 secondary schools in Montreal, Canada. The primary objective is to describe the natural course of ND in relation to cigarette smoking. Over half (55.4%) of eligible students participated; the low response related, in part, to a labour dispute that resulted in some teachers' refusing to collect consent forms. Participants provided assent and parents/guardians provided signed informed consent. Questionnaire data were collected every 3 months during the 10-month school year over a 5-year follow-up period until participants completed secondary school, for a total of 20 cycles[11]. The study received ethics approval from the Montreal Department of Public Health Ethics Review Committee, the McGill University Faculty of Medicine Institutional Review Board and the Ethics Review Committee at the CRCHUM.

### Study variables

Time of initiation of daily cigarette smoking was identified using data collected in a past 3-month recall of cigarette use[12] completed in each cycle. The recall included one item for each of the three months preceding questionnaire administration, which measured number of days on which the participant had smoked cigarettes during that month, and one item for each month that measured number of cigarettes smoked per day on average during that month. Three month test-retest reliability for these two items was very good[13]. If participants checked that they had smoked cigarettes on all 30 days in any of the past three months covered in each cycle, they were categorized as daily cigarette smokers (as of that cycle). Initiation of daily smoking was considered to have occurred during the cycle in which the participant reported smoking daily for the first time.

Seven prognostic indicators were selected based on their association with the initiation of daily smoking, as previously assessed in the NDIT cohort[10] and on the feasibility of collecting accurate data from youth in a clinical setting as indicated by features such as clarity and simplicity of the question to be asked, and ease and rapidity of assessment. Specifically, these included sex, lifetime smoking history (ever, never), ever felt like you really need a cigarette (no, yes), parent(s) smoke (no, yes), sibling(s) smoke (no, yes), friends smoke (no, yes), and alcohol use (never, yes).

Lifetime smoking history was measured in two items: (i) "Have you ever IN YOUR LIFE smoked a cigarette, even just a puff (drag, hit, haul)?" Response choices included no; yes, 1 or 2 times; yes, 3 or 4 times; yes, 5-10 times; and yes, more than 10 times; and (ii) "During the past 3 months, how often did you smoke a cigar or cigarillo?" Response options included never, a bit to try, once or a couple of times a month, once or a couple of times a week, and every day. Participants were categorized as an "ever smoker" if they had a positive response to either item.

"Need a cigarette" was measured in a single item: "How often have you felt like you really need a cigarette?" The four response choices included never, rarely, sometimes, and often. For analysis, responses were recoded into no (never) and yes (rarely, sometimes, often).

Parental smoking was measured by: "Does your father currently smoke cigarettes?" and "Does your mother currently smoke cigarettes?" with response options including no and yes (for each parent). For analysis, a new variable, "parent smoking", was created with response options including no (neither parent smoked) and yes (one or both parents smoked).

"Sibling smoking" was measured by "You have **□ **sisters who smoke cigarettes" and "You have **□ **brothers who smoke cigarettes". Participants were instructed to write the number of sisters/brothers who smoke in the box. If they had no sisters/brothers who smoked, they were instructed to write 0 in the box. For analysis, responses were recoded to no (no sibling smokes) and yes (one or more sibling smokes)

"Friends smoking" was measured by "Now think about your friends. How many of the people whom you usually hang out with smoke cigarettes?" The five response options included none, a few, about half, more than half, most or all. For analysis, responses were recoded into none or a few or more (a few, about half, more than half, most or all).

"Alcohol use" was measured by "During the past 3 months, how often did you drink alcohol?" Response options included never, a bit to try, once or a couple of times a month, once or a couple of times a week, and every day. For analysis, responses were recoded into no (never) and yes (a bit to try, once or a couple of times a month, once or a couple of times a week, and every day).

Mother's education was measured by presenting the respondents with the following five response options: did not finish high school, high school graduate, vocational, technical school, CEGEP, and university.

### Data analysis

The database to study the 1-year risk of daily smoking was created in five steps: (i) observations were divided into four consecutive 1-year waves, each including five data collection cycles (i.e., 1-5, 5-9, 9-13, 13-17); (ii) we determined if participants had initiated daily smoking within each 1-year wave; (iii) if, at the beginning of a 1-year wave, the participants had been categorized as a daily smoker, he/she was removed from that wave and all subsequent 1-year waves; (iv) data on the covariates were drawn from the "baseline" cycle within each wave. (v) data for all participants for all 1-year waves up to and including the cycle in which participants initiated daily smoking or follow-up ended, were pooled across participants and waves. We used the method of multiple imputation to deal with missing values of the covariates. Specifically, we carried out multiple imputation by chained equations with Gibbs sampling using the MICE package available in R[14]. Twenty-five imputation models were run, which included daily smoking, the covariates representing the seven prognostic indicators, and mother's education, which was included as an indicator of socio-economic status due to its potential to be an important determinant of non-response and/or other sources of missingness.

A second database was created to compute the 2-year risk of becoming a daily smoker by subdividing observations into two consecutive 2-year waves, which each included nine cycles (i.e., 1-9, 9-17). The steps to create this second database were analogous to those described above.

Multivariable logistic regression analyses were used to estimate regression parameters, as well as statistics and indicators assessing the model goodness-of-fit and predictive ability. Separate models were fitted for 1-and 2-year risk analyses. The dependent variable was represented by the indicator of initiation of daily smoking over the relevant risk period, and the independent variables were represented by the seven prognostic indicators. We tested potential interactions between the independent variables by adding pair-wise product terms between them to the "main effects only" model to check if any given product term necessitated inclusion. However, none was found to be statistically significant, so that the "main effects only" models were retained as the final models. The description of specific patterns of missingness is provided in Tables 1 and 2

**Table 1:** Patterns of missingness in the 1-year risk analysis.

Number of observations	Gender	Age	Ever smoked	Daily smoker	Friends smoke	Ever felt like need a cigarette	Drink alcohol	Parents smoke	Siblings smoke	Mother's education
2673	+	+	+	+	+	+	+	+	+	+

51	+	+	+	+	+	+	+	-	+	+

101	+	+	+	+	+	+	+	+	-	+

7	+	+	+	+	-	+	+	+	+	+

19	+	+	+	+	+	-	+	+	+	+

29	+	+	+	+	+	+	-	+	+	+

2	+	+	+	-	+	+	+	+	+	+

499	+	+	+	+	+	+	+	+	+	-

6	+	+	+	+	+	+	+	-	-	+

1	+	+	+	-	+	-	+	+	-	+

2	+	+	+	+	+	+	-	+	-	+

1	+	+	+	+	+	+	+	+	-	+

17	+	+	+	+	+	+	+	-	+	-

27	+	+	+	+	+	+	+	+	-	-

1	+	+	+	+	-	+	+	+	+	-

1	+	+	+	+	+	-	+	+	+	-

6	+	+	+	+	+	+	-	+	+	-

2	+	+	+	-	+	+	+	+	+	-

1	+	+	+	+	+	-	+	-	+	-

1	+	+	+	+	+	-	+	+	-	-

1	+	+	+	-	+	+	+	+	-	-

6	+	+	+	+	-	-	-	-	-	+

1	+	+	-	+	-	-	-	-	-	+

9	+	+	+	+	-	-	-	-	-	-

3	+	+	-	+	-	-	-	-	-	-

+ indicates non-missing

- indicates missing

**Table 2:** Patterns of missingness in the 2-year risk analysis.

Number of observations	Gender	Age	Ever smoked	Daily smoker	Friends smoke	Ever felt like need a cigarette	Drink alcohol	Parents smoke	Siblings smoke	Mother's education
1238	+	+	+	+	+	+	+	+	+	+

29	+	+	+	+	+	+	+	-	+	+

13	+	+	+	+	+	+	+	+	-	+

7	+	+	+	+	-	+	+	+	+	+

7	+	+	+	+	+	-	+	+	+	+

13	+	+	+	+	+	+	-	+	+	+

3	+	+	+	-	+	+	+	+	+	+

219	+	+	+	+	+	+	+	+	+	-

1	+	+	+	+	+	+	+	-	-	+

8	+	+	+	+	+	+	+	-	+	-

4	+	+	+	+	+	+	+	+	-	-

1	+	+	+	+	-	+	+	+	+	-

3	+	+	+	+	+	+	-	+	+	-

14	+	+	+	+	-	-	-	-	-	+

1	+	+	+	-	-	-	-	-	-	+

6	+	+	+	+	-	-	-	-	-	-

3	+	+	+	-	-	-	-	-	-	-

+ indicates non-missing

- indicates missing

Potential model overfitting (which could result in the prognostic indicators appearing more discriminating than they actually are) was addressed in bootstrap-based cross-validation (relying on 10,000 replication samples with replacement taken from the analytic dataset)[15]. This allowed us to correct the overfitting bias by applying correction factors (i.e. "shrinkage") to the regression coefficients estimated by the "naïve" logistic models so as to derive their bias-corrected counterparts[16,17]. Specifically, this was carried out as follows. For each of the 10,000 bootstrap samples, the logistic regression model was fitted, producing 10,000 sets of estimated regression coefficients. These were then combined with realizations of the corresponding prognostic indicators to produce 10,000 linear predictor values. Next, logistic regressions were fitted with the linear predictor serving as the only independent variable, producing 10,000 sets of estimated regression coefficients: B_0 _(i.e. the intercept) and B_1 _(i.e. the slope). The 10,000 slope values were then averaged to produce the value of the "shrinkage" factor. The overfitting-corrected regression coefficients were obtained by multiplying the regression slope coefficients from the "naïve" model by the "shrinkage" factor.

We assessed goodness-of-fit of the overfitting-corrected logistic models' by comparing the observed versus expected numbers of outcome events within risk strata, and by carrying out the Hosmer-Lemeshow test[18]. Further, we examined the models' predictive ability by calculating the maximum-rescaled R^2^[19] and the c-statistic[20]. Finally, we assessed the degree of discriminating informativeness of the fitted logistic models (i.e. the extent to which the models are able to risk-stratify) as follows. First, the variance in outcome event probability estimates that would be provided by a hypothetical perfect regression model was calculated as the variance of the distribution of the actual outcome events in the study sample (because a perfect model would produce the probability estimates of 0 for all individuals who would not experience the outcome event during the risk period and the probability estimates of 1 for those who would). Second, the variance in the outcome events' probability estimates provided by the actual fitted models was estimated. The ratio of the latter estimate of variance to the former thus provides a measure of the discriminating informativeness of the actual fitted model relative to the hypothetical perfect model. This measure thus ranges between 0 and 1, with the ratio equal to 0 corresponding to a totally non-informative model and the ratio equal to 1 corresponding to a perfect model.

The regression coefficients estimated in the overfitting-corrected logistic regression models were converted into user-friendly tables, to facilitate their application in practice. All analyses were conducted using SAS v9.13.

## Results

A total of 3467 observations contributed by 1115 individuals with at least some observed values for at least one survey in a given wave were available in the 1-year risk database; the 2-year risk database included 1570 observations contributed by 1004 individuals. Participants in the 1- and 2-year risk databases were similar in terms of the covariates investigated, with the exception that 53% of participants in the 1-year risk database reported that a few or more of their friends smoked, compared to 46% of participants in the 2-year risk database (Table 3). The overall risk of becoming a daily smoker was 6.2% and 12.5% over one- and two-year follow-up intervals, respectively.

**Table 3:** Baseline characteristics of participants, NDIT 1999-2005.

Characteristics	1-year risk analysis (n = 3467)*	2-year Database (n = 1570)†
Male, %	49.4	48.0
Age (years), mean (sd‡)	14.0 (1.1)	13.5 (1.0)
Felt like you really need a cigarette, %	15.5	14.7
Parent(s) smoke, %	29.5	29.8
Sibling(s) smoke, %	17.6	15.7
A few or more friends smoke, %	53.0	45.9
Alcohol use, %	48.1	45.6
Had previously smoked, %	40.6	35.4

* Includes up to four observations per participant.

† Includes up to two observations per participant.

‡ Standard deviation

In the 1-year risk analysis, the overfitting-corrected logistic regression coefficients allowed the calculation of the logit (L) of the probability of initiation of daily smoking as follows: L = -1.15264-0.3161X_1 _+ 1.4954X_2 _+ 0.4042X_3 _+ 0.4834X_4 _+ 0.8376X_5 _+ 0.2935X_6 _+ 1.8216X_7_. In the 2-year risk analysis, the estimated function was: L = 3.2395-0.5382X_1 _+ 1.0600X_2 _+ 0.8577X_3 _+ 0.4959X_4 _+ 0.6597X_5 _+ 0.3002X_6 _+ 1.6481X_7_. The variables X1-X7 represented the prognostic indicators as follows: X_1_: age (years), X_2_: Felt like you really need a cigarette, X_3_: Parent(s) smoke, X_4_: Sibling(s) smoke, X_5_: Friends smoke, X_6_: Alcohol use, X_7_: Ever smoked. Based on the estimated value of L, the probability, or risk, of initiation of daily smoking is calculated according to the logistic transformation: P = 1/(1+e^-L^).

Examination of the distribution of five arbitrary risk categories and the "observed" risk according to the fitted models, suggests reasonably good fit and predictive ability of both the 1-year and 2-year models (Table 4). Specifically, for both models "observed" risk values were close to expected values based on the model-based risk estimation. Further, only 12.9% of participants fell into the 1-year risk category of >5% but ≤10% (i.e. the category that comprises the overall risk of 4.3%), while 56.7% and 10.6% fell into the lowest (i.e. 0-2%) and highest (i.e. > 20%) risk categories, respectively. Only 13.9% of participants fell into the 2-year risk category of >10% but ≤20% (i.e. the category that comprises the overall risk estimate of 12.6%); 14.8% and 20.2% fell into the lowest (i.e. 0-2%) and highest (i.e. >20%) risk categories, respectively. In the 1-year risk model, the p-value for the Hosmer-Lemeshow goodness-of-fit test was 0.71, the c-statistic was 0.87, the maximum-rescaled R^2 ^was 0.31, and the ratio of the actual to theoretically maximum variance in risk estimates was 0.18. In the 2-year risk model, the corresponding values were 0.60, 0.85, 0.33, and 0.18, respectively. The average shrinkage factor values across the 25 multiple imputation sets were 0.99 for both the 1-year and 2-year risk analyses. Thus, the statistical indicators for formal assessment of model performance are consistent with good fit and predictive ability.

**Table 4:** Distribution of risk categories and "observed" (i.e. empirical) risk within them, based on the multivariable logistic models of the 1- and 2-year risk of becoming a daily smoker, NDIT 1999-2005.

	1-year risk analysis		2-year risk analysis	
	
Risk category	% of the sample	"Observed" risk	% of the sample	"Observed" risk
0% but ≤2%	56.7	0.6%	14.8	1.3%
>2% but ≤5%	12.9	4.3%	35.8	2.4%
>5% but ≤10%	12.7	7.3%	15.2	9.5%
>10 but ≤20%	7.1	13.6%	13.9	15.9%
>20%	10.6	32.0%	20.2	38.7%

Tables 5 and 6 present the results of the statistical models converted into points to facilitate the assessment of the 1- and 2-year risk of becoming a daily smoker, respectively. By way of example, according to Table 5, a 12-year old (87 points), who has reported prior smoking (72 points), whose parents smoke (16 points) but not his siblings or friends (0 point), who does not drink alcohol (0 points) but responds positively when asked if (s)he ever felt like having a cigarette (59 points) accumulates 234 points. According to Table 5, he/she has a risk of approximately 23% of initiating daily smoking in the next 1-year period. According to Table 6, an 11-year-old (100 points), who has never smoked or drunk alcohol (0 points), but whose parents (14 points), siblings (11 points), and friends smoke (15 points), and who has felt like smoking a cigarette (25 points) accumulates 175 points. His/her risk of initiating daily smoking in the next 2 years is approximately 63%.

**Table 5:** Assessment of the 1-year risk of initiating daily smoking.

	Points		Enter and add up total points
	**No**	**Yes**	

Ever smoked	0	72	

Parents smoke	0	16	

Siblings smoke	0	19	

Ever felt like need a cigarette	0	59	

Drink alcohol	0	12	

Friends smoke	0	33	

Age (years)			

11	100		

12	87		

13	75		

14	62		

15	50		

16	37		

17	25		

18	12		

19	0		

TOTAL POINTS			

Find the risk corresponding to the total number of points* Interpolation is required if exact total number of points is not presented.			

**TOTAL POINTS**		**1-Year Risk (%)**	

0		0	

100		1	

160		4	

200		12	

220		17	

240		25	

260		40	

280		48	

300		60	

310		67	

* All the values are rounded.

**Table 6:** Assessment of the 2-year risk of initiating daily smoking.

	Points		Enter and add up total points
	**No**	**Yes**	

Ever smoked	0	38	

Parents smoke	0	14	

Siblings smoke	0	11	

Ever felt like need a cigarette	0	25	

Drink alcohol	0	7	

Friends smoke	0	15	

Age (years)			

11	100		

12	87		

13	75		

14	62		

15	50		

16	37		

17	25		

18	12		

19	0		

TOTAL POINTS			

Find the risk corresponding to the total number of points*. Interpolation is required if exact total number of points is not presented.			

**TOTAL POINTS**		**2-Year Risk (%)**	

0		0	

100		6	

120		14	

140		28	

160		48	

180		66	

200		83	

210		89	

* All the values are rounded.

## Discussion

Although tobacco use may be the most important long-term threat to the health of their patients, smoking prevention counselling remains the exception rather than the norm among many pediatricians and other health professionals who interact regularly with children and adolescents. (7) Noting these sub-optimal practices, the American Academy of Pediatrics and other professional societies have strongly recommended the introduction of clinical smoking prevention strategies targeting youth.

The reasons why physicians and other health care practitioners fail to offer smoking prevention counselling to their young patients are not well understood. They may feel less urgency about smoking prevention because few of their young patients smoke and those who do smoke, do so only sporadically or infrequently, and therefore are not yet at high risk of smoking-related health problems. Alternatively, health professionals may believe that counselling is outside their role or that counselling is ineffective for pediatric patients and that prevention of injuries or obesity is more important in this age range. Finally they may lack knowledge on community resources to which to refer their patients for more intensive intervention and follow-up.

Because physicians and other health care professionals have limited time to devote to prevention, they need to prioritize their counselling to maximize impact. If it were possible to rapidly identify youth at high risk of becoming sustained long-term smokers, they could either offer more intensive counselling or refer these patients to specialized community resources.

The user-friendly prognostic tool developed herein can be used in health care practice to identify youth at high risk of initiation of daily smoking over a one- or two-year time period. Points are added based on age and yes/no answers to six simple questions. The total number of points is then converted into the one- or two-year probability of becoming a daily smoker.

Because there is no clinical consensus or guidelines defining what "high" risk of initiating daily smoking actually is, physicians and their patients will need to rely on their judgement and value systems to define "high risk" and "low risk" on an individual basis, to decide when intervention is warranted. These decisions may be influenced by the availability of practice-based or community resources for smoking prevention, prevailing social norms, and physician preferences and comfort in providing counselling.

The degree of applicability of the developed prognostic tool across populations remains to be established. They will need to be tested in different settings to assess replicability and external validity before they can be recommended for general use. Still, we believe that their performance should be sufficiently robust because most items included in the models are well-established determinants of youth smoking behaviour[10]. In addition, overfitting-corrected measures of goodness-of-fit and predictive ability of our models suggest adequate validity and discriminating informativeness. The prognostic indicators investigated were limited to those assessed in NDIT. However, the data collected in NDIT were based on an exhaustive literature search of the most important determinants of cigarette smoking and represent characteristics which can be assessed easily and rapidly (within 1-2 minutes) in a clinical (or even non-clinical) setting. One item, intention to smoke, that was not collected in the NDIT could potentially contribute extra information in assessing the risk of initiation of daily smoking. Future studies should investigate the added value of including this item into a prognostic tool such as ours. Finally, because participants were aged 11-19 years, the results may not be generalizable to individuals outside this age range.

## Conclusion

This prognostic tool is ultimately useful only if there are effective youth tobacco control interventions. More research into prevention and cessation interventions targeting paediatric populations is needed to reduce smoking prevalence. The use of prognostic tools to identify high risk youth in combination with effective clinical and community-based intervention and public policy may eventually contribute significantly to reducing tobacco use among youth.

## Competing interests

The authors declare that they have no competing interests.

## Authors' contributions

IK participated in the conception and design of the study, interpretation of the data and drafted the manuscript. GP contributed to the conception and design of the study, interpretation of the data and participated in the drafting of the manuscript. ML contributed to interpretation of the data and participated in the drafting of the manuscript. ED participated in the design and coordination of the study and contributed to interpretation of the data. JOL conceived of and designed the study, obtained the funding, participated in its coordination, contributed to interpretation of the data, and participated in the drafting of the manuscript. All authors read and approved the final manuscript.

## Funding

This work was supported by the Canadian Cancer Society (grant numbers 010271, 017435).

## Pre-publication history

The pre-publication history for this paper can be accessed here:

http://www.biomedcentral.com/1471-2431/11/70/prepub
